# Differential Functionality of Right and Left Parietal Activity in Controlling a Motor Vehicle

**DOI:** 10.3389/fnsys.2016.00106

**Published:** 2016-12-27

**Authors:** Justin R. Brooks, Javier O. Garcia, Scott E. Kerick, Jean M. Vettel

**Affiliations:** ^1^Human Research and Engineering Directorate, US Army Research LaboratoryAdelphi, MD, USA; ^2^Department of Psychological and Brain Sciences, University of CaliforniaSanta Barbara, CA, USA; ^3^Department of Bioengineering, University of PennsylvaniaPhiladelphia, PA, USA

**Keywords:** driving, attention, heading error, parietal lobe, alpha rhythm, lane deviation, steering wheel

## Abstract

Driving a motor vehicle is an inherently complex task that requires robust control to avoid catastrophic accidents. Drivers must maintain their vehicle in the middle of the travel lane to avoid high speed collisions with other traffic. Interestingly, while a vehicle’s lane deviation (LD) is critical, studies have demonstrated that heading error (HE) is one of the primary variables drivers use to determine a steering response, which directly controls the position of the vehicle in the lane. In this study, we examined how the brain represents the dichotomy between control/response parameters (heading, reaction time (RT), and steering wheel corrections) and task-critical parameters (LD). Specifically, we examined electroencephalography (EEG) alpha band power (8–13 Hz) from estimated sources in right and left parietal regions, and related this activity to four metrics of driving performance. Our results demonstrate differential task involvement between the two hemispheres: right parietal activity was most closely related to LD, whereas left parietal activity was most closely related to HE, RT and steering responses. Furthermore, HE, RT and steering wheel corrections increased over the duration of the experiment while LD did not. Collectively, our results suggest that the brain uses differential monitoring and control strategies in the right and left parietal regions to control a motor vehicle. Our results suggest that the regulation of this control changes over time while maintaining critical task performance. These results are interpreted in two complementary theoretical frameworks: the uncontrolled manifold and compensatory control theories. The central tenet of these frameworks permits performance variability in parameters (i.e., HE, RT and steering) so far as it does not interfere with critical task execution (i.e., LD). Our results extend the existing research by demonstrating potential neural substrates for this phenomenon which may serve as potential targets for brain-computer interfaces that predict poor driving performance.

## Introduction

Everyday visual-motor tasks involve complex interactions of multiple sensory signals that are converted into descending motor commands and translated to movement. This concept has been formalized from a number of frameworks including sensorimotor transformations (Pouget and Snyder, 2000) and motor (feedback) control theory (reviewed in Shadmehr et al., 2010). Within feedback theory, one of the major control challenges that has emerged arises from the large degrees of freedom and redundancies for a given visual-motor movement. For example, a reaching movement that places the hand at a particular location can be achieved with variable orientations of the shoulder, elbow and wrist joints. To address this issue, a theoretical framework emerged in which these degrees of freedom are allowed to freely vary so long as they do not interfere with the primary objective of acquiring the desired end state of the movement (reviewed in Todorov and Jordan, 2002; Scott, 2004). Here, we examine how these control parameters may be monitored by the brain to ensure successful task execution.

To contend with superfluous redundancies for a visual-motor movement, the brain must monitor perceptual input and sensory feedback to efficiently execute visual-motor tasks, and previous research posits that attention may give priority to behaviorally relevant actions (Wulf et al., 2001). Using electroencephalography (EEG), a relationship between alpha band power and attentional state has been identified in the seconds preceding a stimulus (reviewed in Hanslmayr et al., 2011). Studies have suggested that this prestimulus alpha activity is correlated with gain and processing of the visual stream, and prestimulus alpha may be subject to top-down visuospatial attention mechanisms (Engel et al., 2001; van Dijk et al., 2008; Gould et al., 2011; Sonnleitner et al., 2012; Gilbert and Li, 2013). Further investigations have suggested that increased alpha power is associated with the inhibition of distracting sensory information (i.e., functional inhibition; Jokisch and Jensen, 2007) and gating of sensory input (Jones et al., 2010; Klimesch et al., 2011; Sonnleitner et al., 2012). Consequently, fluctuations in alpha may reflect changes in visuospatial attention that are related to tracking the relevant sensory parameters needed to successfully execute a visual-motor task.

In this experiment, we examine the interactions between visuospatial attention and visual-motor movements during a highly learned naturalistic task, driving a motor vehicle. Behavioral studies have suggested that a vehicle’s heading error (HE) is one of the primary parameters used by humans to control the steering wheel (Hildreth et al., 2000; Wallis et al., 2002; Cloete and Wallis, 2009). Complementary research has modeled the steering of a vehicle as a feedback control system and demonstrated the importance of visual information for this control (Donges, 1978; Hess and Modjtahedzadeh, 1990; Salvucci and Gray, 2004). While there is behavioral evidence to support the relevance of HE, several EEG studies have identified the importance of additional driving performance metrics, including lane deviation (LD), lane crossing incidence, and reaction time (RT). These neuroimaging analyses have demonstrated that increasing levels of alpha (8–13 Hz) power are associated with decrements in driving performance (Horne and Baulk, 2004; Lin et al., 2005, 2010; Huang et al., 2009; reviewed in Lin et al., 2012). In addition to these frequency effects in EEG, several functional magnetic resonance imaging (fMRI) studies have shown that activity in parietal brain regions is associated with vehicle heading, obstacle avoidance and path estimation from optic flow (Walter et al., 2001; Horikawa et al., 2005; Field et al., 2007; Spiers and Maguire, 2007). Taken together, these studies suggest that the parietal cortex may monitor vehicle dynamics to maintain control during driving, but it is unclear what driving metric best reflects the relationship between parietal activity and subsequent visual-motor performance.

Here, we recorded EEG while participants performed a simulated continuous driving task. They were required to make corrective steering movements in response to periodic perturbations that simulated a wind gust and pushed the vehicle off course. The EEG data were decomposed using independent component (IC) analysis, and a cluster of components in both right and left parietal cortex were used in a generalized linear model (GLM) to examine relationships between driving performance and fluctuations in alpha activity as an index of visuospatial attention. We hypothesized that periods of relatively higher alpha power in parietal regions prior to the onset of the perturbation would show a strong relationship with four metrics of driving performance (LD, HE, RT and steering response). Furthermore, we expected that differential parietal activity with these four metrics would reveal how the brain imparts control on a moving vehicle in a continuous, naturalistic task. Our results found pre-stimulus alpha power in the right parietal cortex was related to variability in LD, while pre-stimulus alpha in left parietal was linked to HE, RT and steering. These findings suggest that the brain uses a dynamic control strategy to maintain central task requirements, and this strategy relies on fluctuations in alpha power which likely reflects dynamic attention allocation needed to track sensory parameters relevant for successfully executing a visual-motor task.

## Materials and Methods

### Participants

The participants were neurologically intact, healthy, adult right-hand- and right-eye-dominant males (*N* = 10; age range 27–39). The voluntary informed consent of the participants was obtained following US Army human use regulations approved by the Army Research Laboratory Institutional Review Board (32 CFR 219 and DoDI 3216.02).

### Experimental Design

**Figure 1:**
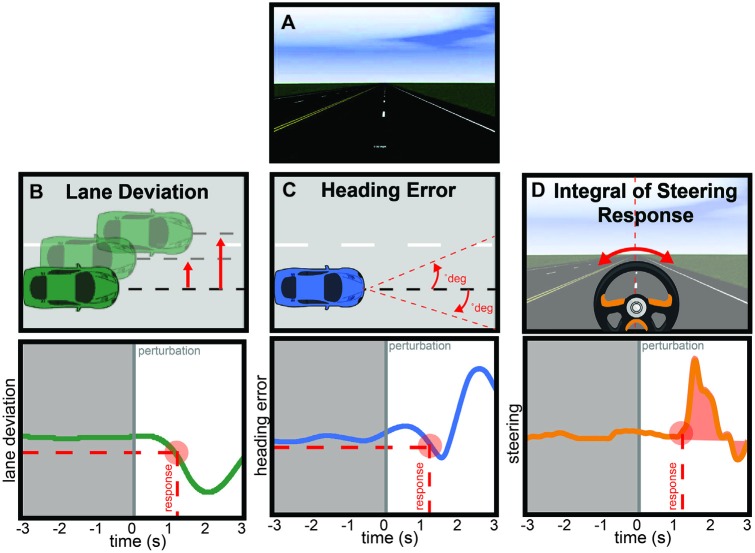
**(A)** Screenshot from the driving simulator.** (B)** Lane deviation (LD) was computed as the absolute value of the distance from the middle of the travel lane to the middle of the vehicle (top) at the time that the participant initiated a steering response (bottom, red circle) in response to the perturbation (bottom, time 0 s). **(C)** Heading error (HE) was computed as the absolute value of the angular difference between the vehicle’s current course and an infinitely straight line from the middle of the vehicle (top) at the time that the participant initiated a steering response (bottom, red circle) in response to the perturbation (bottom, time 0 s). **(D)** Integrated steering response (ISR) was computed as the area underneath the steering wheel time series from the time of response to the end of the epoch (red shaded region). **(B–D)** Gray shaded region denotes the time over which alpha spectral power was calculated.

The driving simulator consisted of a 24″ monitor, computer speakers, and a steering wheel with accelerator and brake foot pedals situated in an acoustically-attenuated room. Simulation of the visual and audio task environment was developed and rendered in real-time using SimCreator (Real Time Technologies, Ann Arbor, MI, USA). The simulation environment consisted of a long straight four-lane highway with minimal scenery (highway, roadside and horizon) and no traffic or changes in roadside stimuli, with the exception of a speed limit sign that was presented approximately every 10 min (Figure 1A). Vehicle and environmental sounds were muted, except for a tone that activated if the participants drove beyond the shoulder of the right-most lane (off the road) or across the median of the highway (into oncoming traffic lane).

The participants first acclimated to the simulation environment and vehicle dynamics during a 15-min drive where the vehicle speed was constant at 45 mph and the participants only had to control steering. Following a 5-min break, participants drove for 45 min. They were instructed to steer the vehicle to maintain the vehicle in the center of the right-most lane and adjust their speed in accordance to speed limit signs (25 mph or 45 mph). The participants were informed that a perturbation force would occasionally occur, causing the vehicle to veer left or right in a manner similar to the effects experienced when a gust of wind crosses a real vehicle (e.g., see Thiffault and Bergeron, 2003; Lin et al., 2005). They were asked to correct the vehicle by steering it back to the center as soon as possible in the event of a perturbation. The environment included perturbations that were randomly distributed in right and left directions at a rate of one per 8 s (+0–2 s) with the criteria that participants maintain the vehicle within the right-most lane boundaries for at least 8 s before the next perturbation would occur (Lin et al., 2005). The perturbation force was canceled once participants turned the steering wheel 4° in the compensatory direction. If they did not respond within a few seconds, the force would angle the vehicle into the left or right side boundaries and trigger the warning tone. Every 15 min, the experimenter talked to the participant over an intercom and asked the participant to verbally report their sleepiness rating from 1 (extremely alert) to 9 (extremely sleepy, fighting sleep) when prompted. In the present article, our analysis focuses only on the data during the 45 min drive.

### Behavioral Analysis

Although the participants drove continuously, three metrics were computed around each perturbation event. The LD was measured in meters and computed as the absolute value of the vehicle’s position from the center of the participant’s traveling lane (Figure 1B, top). The HE was measured in degrees and computed by determining the angular difference between the vehicle’s current course and an infinitely straight line that is parallel to the road edge (Figure 1C, top). Finally, the integrated steering response (ISR) was the integral of the steering wheel position from the time of response to 3 s after the perturbation started (Figure 1D, top). Each calculation occurred when the participant’s steering wheel response was ≥4° in the direction counteractive to the perturbation force. The time elapsed between the initiation of the perturbation and the response determined the RT (circle, Figures 1B–D, bottom). Simple *t*-tests showed no differences (all *p* > 0.1) in the steering responses, HE, RT or LD as a function of leftward or rightward perturbations. Therefore, in our subsequent analyses, we collapsed across right and left perturbations, and this decision did not impact the brain data analysis which examined the 3 s before perturbation onset. Finally, to compare measures across subjects, each driving performance measure was *z*-scored separately within each subject so that across subject variability was normalized.

### Electroencephalography (EEG) Acquisition

EEG data were acquired using a BioSemi system (Amsterdam, Netherlands). Continuous recordings were sampled at 2048 Hz and acquired from 64 standard scalp locations (10–10 system; Chatrian et al., 1988) referenced online to the Common Mode Sense (CMS) electrode. Six additional channels were also recorded with online reference to CMS: left/right mastoids (M1/M2), two horizontal (left/right; HEOL/HEOR) and two vertical (upper/lower right eye; VEOU/VEOL) electro-ocular electrodes. EEG data were synchronized with the driving simulator CPU’s via an Arduino-based system.

### EEG Preprocessing

All signal processing was applied using EEGLAB (ver 11.0.0.0b; Delorme and Makeig, 2004,^1^) and in-house code using Matlab (R2012a) on a 64-bit Linux operating system. Following established preprocessing procedures (Onton et al., 2006), all electrodes (64 EEG + 4 EOG) were first re-referenced to the average of the two mastoid electrodes (M1 and M2) before being bandpass filtered between 1 Hz and 50 Hz using a zero-phase finite impulse response filter (EEGLAB function *pop_eegfilt*). The EEG data for each participant was epoched into non-event-locked, adjacent (non-overlapping), 500-ms windows, and automatic artifact-rejection algorithms were applied to the epoched data using EEGLAB function *pop_rejmenu* with the following rejection criteria: (1) amplitude threshold (> ±100 μV); (2) joint probability (>5 SD); (3) abnormal trends (max slope 75 μV/500-ms epoch; R-squared limit 0.3); and (4) kurtosis (>5 SD). Finally, we visually inspected all epochs and confirmed automatically-tagged epochs containing artifacts or manually accepted or rejected epochs if incongruent with visual inspection.

### EEG Analysis

We applied infomax independent component analysis (ICA; Bell and Sejnowski, 1995; Lee et al., 1999) and obtained spatially fixed, maximally temporally IC processes (Makeig et al., 1996) for each participant. A single model decomposition was used with default extended-mode training parameters (EEGLAB function *runica*). We then obtained dipoles for all ICs using EEGLAB plugin dipfit and selected features for clustering ICs using EEGLAB function *pop_preclust* (spectra = 1, ERSP = 1 and dipole models = 10). By default this technique uses the Four-Shell spherical head model. Clustering was performed using the k-means algorithm (EEGLAB function *pop_clust*) with number of clusters set to 19 and outliers set to >2.5 standard deviation from cluster centers.

**Figure 2:**
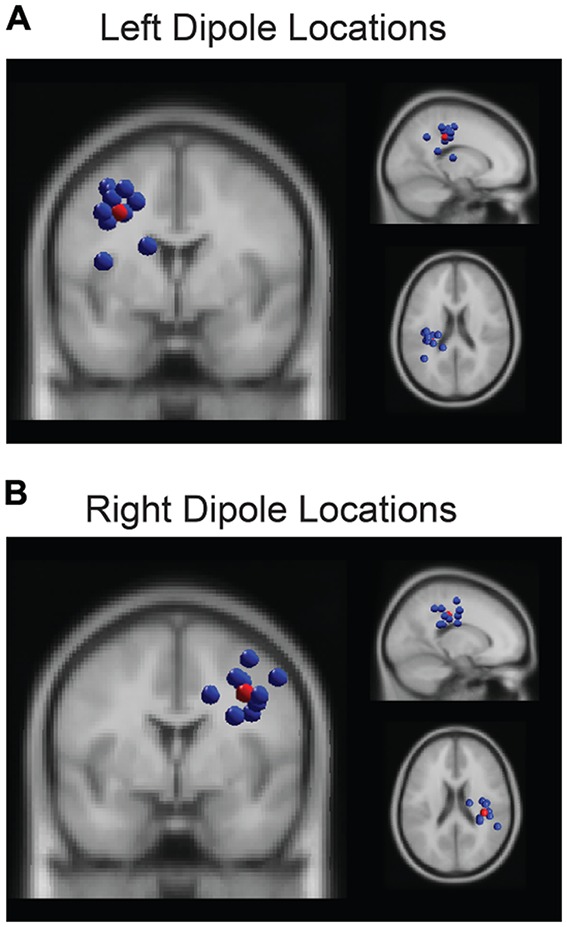
**The k-means clustering algorithm identified two parietal clusters which included one independent component (IC) for each participant in left (A)** and right **(B)** parietal regions. The centroids (red spheres) were located at (−31, −21, 36) and (35, −28, 34) respectively.

Based on our *a priori* hypothesis, our analysis focused on two of the identified clusters in left and right parietal regions. The algorithm assigned 10 total ICs to each of the parietal clusters (Figure 2), including one component per participant whose dipole was proximal to parietal cortex so no manual adjustments were made to the output clusters. For each IC in each parietal cluster, we generated epochs around each perturbation event (−3000 ms to 3000 ms) and *z*-scored the power on a trial-by-trial basis using a wavelet decomposition (Morlet) to obtain average dynamic changes in spectral amplitude within the alpha band (8–13 Hz). This procedure aligns with previous research that demonstrated less sensitivity to noisy trials by using the entirety of the trial epoch period, instead of the traditional pre-stimulus period, when computing spectral power estimates (Grandchamp and Delorme, 2011). This normalization procedure (using the newtimef function in EEGLAB) also ensured common units of standard deviation so that we could directly compare behavior to neural data. The regression analysis, then, focused on the pre-perturbation period and used the averaged *z-scored* values of alpha power from −3000 ms to 0 ms. As confirmatory analyses, we also verified that the inclusion of the post-perturbation period in the normalization procedure did not bias the results by comparing the correlation between pre- and post-perturbation values (*R*^2^ = 0.1 for left hemisphere and *R*^2^ = 0.1 for right), and we did not find significant correlations between the alpha activity during the post-perturbation and any of the four metrics of driving performance (*p* > 0/05).

### Statistical Analysis

We used a GLM to investigate the relationship between driving performance and alpha activity in right (*R*_α_) and left (*L*_α_) parietal dipole IC sources. Four separate models were run for the four driving metrics of interest (Behavior): LD, RT, HE and steering response. We controlled for time-on-task effects that may also modulate alpha activity and changes in vehicle-road kinematics (Otmani et al., 2005; Wascher et al., 2016) by including a parameter for time elapsed from the beginning of the experiment to the time of the trial measured in seconds (Time). In the equation, the * denotes that all main effects, pairwise effects, and three-way interactions were modeled simultaneously.

$$\text{Behavior}\;=\;R_{\text{α}}{\;}^{*}\;L_{\text{α}}{\;}^{*}\;\text{Time}$$

For each GLM, a backward selection procedure was used to select the most parsimonious model that was able to account for the variability in behavioral performance across 1896 trials. First, the model was fit with coefficients for the three main effects (Right, Left and Time), the pairwise effects (Right-Left, Right-Time, Left-Time), the three-way interaction (Right-Left-Time) and the intercept. The coefficients were then sorted by *p*-values, and the coefficient with the largest, insignificant *p-value* (*p* > 0.05) was eliminated from the model. If that coefficient represented a main effect, all higher order interaction terms containing that effect were eliminated. This procedure continued until all remaining coefficients were significant (*p* < 0.05) and identified terms with a significant relationship to the driving behavior, or the procedure terminated when no terms remained in the model which indicated no relationship with the driving behavior. To control for multiple comparisons, we performed false discovery rate correction (Benjamini and Hochberg, 1995). As a confirmatory analysis, we also examined within-subject variability by including a subject-specific term in the regression model, but the direction and significance of the identified coefficients in Table 1 remained the same with only small fluctuations in magnitude.

**Table 1:** **Summary of the generalized linear model (GLM) analysis with values from the final model**.

Behavior	Right	Left	Time	Right:Left	Right:Time	Left:Time	Right:Left:Time
LD	***0.049 (0.05)***	−0.036	0.02	0.008	0.0065	0.042	0.053 (0.02)
HE	−0.005	***0.088 (<0.001)***	***0.11 (<0.001)***	0.027	0.017	−0.002	0.014
Steering	0.013	***0.055 (0.03)***	***0.094 (<0.001)***	0.027	0.009	0.0373	0.044
RT	0.01	***0.09 (<0.001)***	***0.07 (<0.01)***	0.017	−0.02	0.05	0.009

*Each row shows the model results for three driving behaviors: lane deviation (LD), heading error (HE), and integrated steering response (Steering). Each column shows model coefficients and their respective *p*-values in parentheses for the terms in the final model, including alpha power in two parietal clusters (Right and Left) as well as a time on task term (Time). Columns with colons indicate interaction terms. Significant effects retained in the final model are in italicized bold. Terms not included in the final model appear in regular text*.

## Results

In this study, 10 participants performed a simulated continuous driving task for 45 min on an infinitely long straight highway. Periodic perturbations that simulated a wind gust were imposed, resulting in a subsequent LD, HE and required a steering response and associated RT. We hypothesized that periods of relatively higher alpha power in parietal regions prior to the onset of the perturbation would show a strong relationship with these four driving performance metrics and that differential activity may provide insight into how the brain imparts control on a moving vehicle.

### Behavioral Results

The average number of total perturbations across participants: 189, standard deviation (SD): 29. As visualized in Figure 1, we computed four performance metrics around each perturbation event, LD, HE, RT and steering response. These metrics were calculated at the onset of the participant’s steering response to counteract the perturbation force. The average RT across all participants: 1.16 s, SD: 0.46 s; the mean LD: 0.373 m, SD: 0.33 m. The average HE at the time of response: 23.49°, SD: 29.28°; and the average ISR: 6.71°, SD: 4.88°.

### Relationship between Lane Deviation and Parietal Alpha Activity

**Figure 3:**
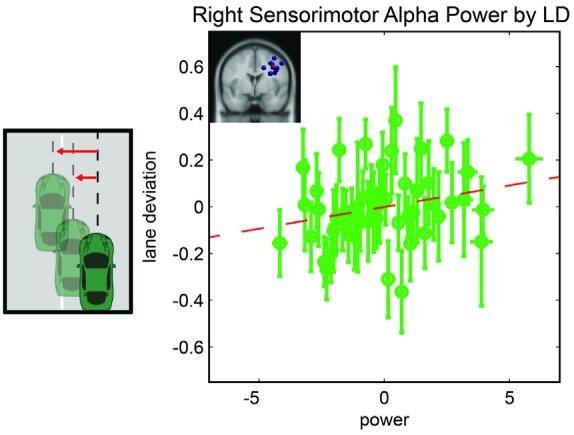
**Resampled data to reflect the relationship between LD and alpha power.** Since subjects had variable numbers of trials, we resampled to 50 trials for each subject. Each point represents the mean across a single resampled trial for all 10 subjects and error bars reflect the standard error of the mean. This was done strictly for visualization purposes the data were not resampled for our statistical analyses. The best fit line is superimposed in red.

We computed a general linear model to examine driving performance as measured by LD as a linear combination of left and right parietal alpha power, time-on-task effects, and the interaction of these variables. In the initial model fit (*F*_(7,1888)_ = 2.6, corrected *p*-value = 0.02), prior to the backward selection procedure, the *p-value* for all terms were larger than 0.1 except for the main effect for right parietal alpha activity and the three way interaction term (see Table 1). After the model selection procedure; however, the only term that remained in the model was the right parietal alpha activity (coefficient = 0.047 with a 95% confidence interval of [0.002–0.09], *p-value* = 0.038). The relationship between right alpha power and LD for all subjects is plotted in Figure 3.

### Relationship between Heading Error and Parietal Alpha Activity

**Figure 4:**
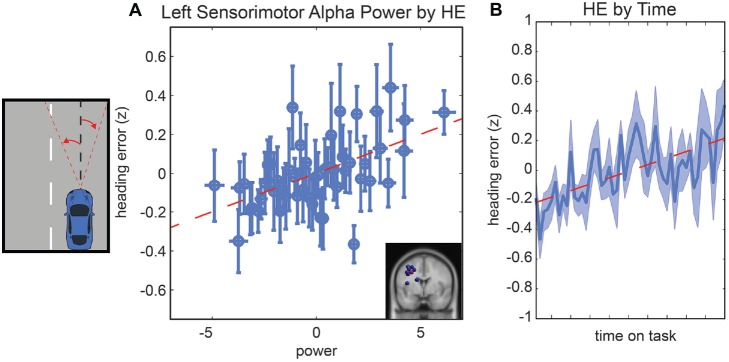
**Resampled data to reflect the relationship between HE and alpha power (A)** and the relationship between HE and time** (B)**. Since subjects had variable numbers of trials, we resampled to 50 trials for each subject. Each point represents the mean across a single resampled trial for all 10 subjects and error bars reflect the standard error of the mean. This was done strictly for visualization purposes the data were not resampled for our statistical analyses. The best fit line is superimposed in red in both plots.

Following the same procedure used for LD, we computed a general linear model to examine driving performance as measured by the vehicle HE as a linear combination of left and right parietal alpha power, time-on-task effects and the interaction of these variables. In the initial model fit (*F*_(7,1888)_ = 6.76, corrected *p*-value < 0.01), prior to the backward selection procedure, the *p-value* for all terms were larger than 0.1 except for the main effect for left parietal alpha activity and the main effect for time-on-task (see Table 1). After the model selection procedure, however, the same two terms remained in the model, left parietal alpha activity (coefficient = 0.09 with a 95% confidence interval of [0.046–0.13], *p-value* < 0.01) and the time-on-task (coefficient = 0.11 with a 95% confidence interval of [0.07–0.16], *p-value* < 0.01). The relationship between left alpha power and HE for all subjects is plotted in Figure 4A, and the relationship between time-on-task and HE for all subjects is plotted in Figure 4B.

### Relationship between Steering Response and Parietal Alpha Activity

**Figure 5:**
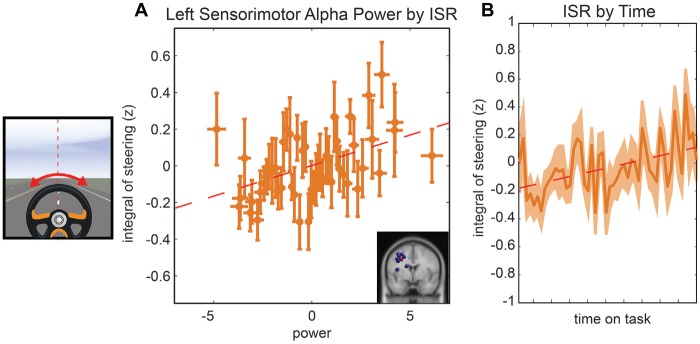
**Resampled data to reflect the relationship between ISR and alpha power (A)** and the relationship between ISR and time **(B).** Since subjects had variable numbers of trials, we resampled to 50 trials for each subject. Each point represents the mean across a single resampled trial for all 10 subjects and error bars reflect the standard error of the mean. This was done strictly for visualization purposes the data were not resampled for our statistical analyses. The best fit line is superimposed in red in both plots.

The last model we examined was the relationship between the ISR, parietal alpha activity, and time. In the initial model fit (*F*_(7,1888)_ = 6.5, corrected *p*-value < 0.01), prior to the backward selection procedure, the *p-value* for all terms were larger than 0.1 except for the main effect for left parietal alpha activity and the main effect for time-on-task (see Table 1). The same two terms remained in the model after the model selection procedure, left parietal alpha activity (coefficient = 0.07 with a 95% confidence interval of [0.052–0.114], *p-value* < 0.01) and the time-on-task (coefficient = 0.11 with a 95% confidence interval of [0.065–0.154], *p-value* < 0.01). The relationship between left alpha power and the ISR for all subjects is plotted in Figure 5A, and the relationship between time-on-task and ISR for all subjects is plotted in Figure 5B.

### Relationship between Reaction Time and Parietal Alpha Activity

**Figure 6:**
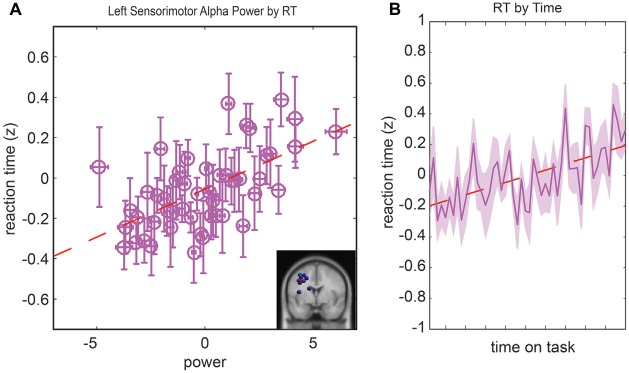
**Resampled data to reflect the relationship between reaction time (RT) and alpha power (A**) and the relationship between RT and time **(B)**. Since subjects had variable numbers of trials, we resampled to 50 trials for each subject. Each point represents the mean across a single resampled trial for all 10 subjects and error bars reflect the standard error of the mean. This was done strictly for visualization purposes the data were not resampled for our statistical analyses. The best fit line is superimposed in red in both plots.

The last model we examined was the relationship between the RT, parietal alpha activity, and time. In the initial model fit (*F*_(7,1888)_ = 5.13, corrected *p*-value < 0.01), prior to the backward selection procedure, the *p-value* for all terms were larger than 0.07 except for the main effect for left parietal alpha activity and the main effect for time-on-task (see Table 1). The same two terms remained in the model after the model selection procedure, left parietal alpha activity (coefficient = 0.1 with a 95% confidence interval of [0.059–0.148], *p-value* < 0.01) and the time-on-task (coefficient = 0.07 with a 95% confidence interval of [0.023–0.113], *p-value* < 0.01). The relationship between left alpha power and the RT for all subjects is plotted in Figure 6A, and the relationship between time-on-task and RT for all subjects is plotted in Figure 6B.

## Discussion

Participants drove a simulated vehicle for 45 min on an infinitely long, straight highway, and periodic perturbations occurred that pushed the vehicle off course and caused a subsequent LD and HE. Participants were required to steer the vehicle back to the center of the lane, and we hypothesized the variability in prestimulus alpha to the perturbation event would relate to variability in driving performance. EEG was decomposed using IC analysis, and a cluster of components in left and right parietal cortex was used to examine the relationship between alpha power and four driving performance metrics: LD, HE, RT and steering response. We uncovered differential relationships in the left and right parietal cluster, revealing that HE, RT and steering response track with activity in left parietal sources while LD links to alpha activity in the right parietal sources. Our findings overall suggest that right parietal activity is related to attentional states that orient the vehicle on the road in support of the central visual-motor task whereas left parietal alpha activity reflects attentional processing that focuses on HE in preparation for minimizing the RT to make corrective steering responses.

### Relationship between Driving Performance and Parietal Alpha Activity

The right parietal component cluster demonstrated a significant, positive relationship with LD. Previous work has demonstrated that activity in right parietal cortex supports visuospatial processing and orienting (Whitehead, 1991; Rushworth et al., 2001; Foxe et al., 2003; Thut, 2006), and additional research has refined its role, suggesting that the right hemisphere is more closely aligned to events with higher temporal predictability, increased arousal, or heightened vigilance (Fernández and Siéroff, 2014). In line with these findings, right parietal alpha activity increased when the participant was slow to correct for the recent wind perturbation and allowed the amount of LD to increase. Increased pre-stimulus alpha power has been linked to decrements in subsequent performance (Hanslmayr et al., 2011), and in the context of driving, it has been interpreted as decreased arousal with impaired vigilance (Lin et al., 2012). Our findings suggest that the right parietal cortex reflects visuospatial processing that is critical for tracking vehicle kinematics for the central task (maintaining a zero LD), and fluctuations in alpha may capture fluctuations in attention that are critical for driving performance. Interestingly, recent clinical work supports this interpretation by demonstrating the patients with right hemisphere lesions have significantly more lane crossing incidents that those with left hemispheric lesions, and furthermore, this study found that left hemisphere patients demonstrated longer RT for braking responses than those with right hemisphere lesions (Park, 2015). These clinical findings nicely complement our results from healthy participants.

The left parietal component cluster demonstrated a significant, positive relationship with HE and steering response. Previous behavioral research has shown that HE is closely related to upcoming steering responses (Hildreth et al., 2000; Field et al., 2007). Our results align well with previous research that suggested that left parietal regions may direct attention for upcoming movements (Rushworth et al., 2001) as well as studies where preparatory attention is modulated by the left hemisphere (Fernández and Siéroff, 2014). Collectively, our current results suggest that left parietal alpha activity relates to processes which monitor the current heading of the vehicle to determine the timing and magnitude of the response for subsequent corrective action.

These results also align with previous research that uncovered differential network activity related to adaptive and stable task control (Dosenbach et al., 2007). Their study uncovered a frontoparietal network that demonstrated error-related activity on a trial by trial basis, and a secondary network that had sustained activity throughout the task. Our results are similar. Activity in the left parietal cluster related to HE, RT and coordinated steering control—all of which were dynamic over the course of the experiment. Conversely, the right parietal region had sustained activity related to the critical task parameter, LD, which did not change over the course of the experiment. These findings suggest that parietal activity may reflect a dynamic control strategy to maintain critical performance when executing a visual-motor task.

### Differential Effects of Time on Task on Driving Performance

Across the models for the four driving performance metrics, LD did not show a time on task effect, while both HE, RT and steering response increased over the course of the long drive. Previous research has suggested that humans are exceedingly vigilant against infractions of the central task requirement (Hockey, 1997; Grier et al., 2003; van der Linden et al., 2003), and in our study, the participants were instructed to keep their vehicle in the center of the traveling lane. In support of this previous literature, our results revealed that LD did not show a main effect of time, indicating that participants successfully minimized performance decrements in their primary task.

In contrast, the models for both HE and steering response revealed a main effect of time. The behavioral coupling between these two driving metrics has been previously shown suggesting that they may be incorporated in a complex feedback control system wherein the HE is perceived and transformed into a steering response. In support of this, behavioral research has found that the HE is more closely linked to steering responses than to LD (Hildreth et al., 2000; Wallis et al., 2002; Cloete and Wallis, 2009; Li and Cheng, 2011). In our study, we extended these results and observed that both driving metrics increase over the duration of the experiment. When construed as a change in the control parameter, the increase in HE over time suggests that the brain permits increased error in this regulatory dimension, but in order to maintain the LD near zero for the central task requirement, the participant utilized larger steering wheel corrections. This paired increase in HE and steering response is analogous to uncontrolled manifold hypotheses (Scholz and Schöner, 1999; Todorov, 2004) wherein a number of different heading/steering corrections over time can lead to a small, near zero LD. Combined, our results suggest that the brain utilizes HE to estimate steering responses, and as time on task effects negatively impact driving performance (Matthews and Desmond, 2002), the increased tolerance for larger HEs leads to a concomitant change to larger steering wheel corrections. This is supported by the observation that RTs increased over the course of the experiment which may suggest that monitoring processes degraded over the course of the experiment and resulted in larger heading responses and subsequent increases in steering responses. Our data cannot be directly interpreted as an explicit feedback control model since we do not model the dynamics of this relationship (e.g., steering responses over time), but this pattern can be interpreted in terms of a feedback control system that allows varying levels of error in control parameters while maintaining accuracy in the central visual-motor task. More specifically, right parietal activity in our study was associated with monitoring of LD (the central, critical task requirement), while left parietal activity associated with feedback control perhaps in the computation of the control signal. Future research can target experimental designs to test these interpretations.

## Future Directions

The main motivation for our work targets a driver error detection system to mitigate accidents that occur due to lapses in attention and visuomotor control. Consequently, we focused on four driving performance metrics that are the most amenable to study a closed loop control problem, but additional measures such as standard deviation of lane position (SDLP) or steering wheel reversal rate (SWRR) could provide interesting insights about driving performance more generally (for a complete review of driving metrics, see Sandberg et al., 2011). Likewise, we narrowed our focus to alpha activity due to its role in attention and strong relationship to subsequent task performance (Hanslmayr et al., 2011); however, it would be interesting to examine the contribution from other frequency bands. Previous work has shown that alpha and theta positively related with incidents (*r* from 0.72 to 0.90; Horne and Baulk, 2004), and preparatory action has been associated with the beta band (Tzagarakis et al., 2015), suggesting an interesting avenue for future driving research. We also only examined the brain activity before the pre-perturbation period when participants were maintaining ongoing driving performance, but there are several, critical cognitive processes that occur after perturbation onset that must capture the ongoing demands of the task in order to execute an efficient and accurate response. Work to capture the dynamics of these processes may also be essential for the successful implementation and adoption of driver error detection systems. Finally, our targeted focus on parietal activity could be expanded to look at additional regions robustly identified in previous driving research (Calhoun et al., 2002; Spiers and Maguire, 2007; Huang et al., 2009) to better delineate the distributed neural substrates that support accurate driving performance. All of these future directions would nicely extend our findings, and with larger sample sizes, the research could also target within-subject analyses using more advanced approaches (e.g., hierarchical mixed effects) in order to better understand how driver error detection system can be adapted to differences between individuals.

## Conclusion

In this realistic, continuous driving task, we have demonstrated differential relationships between neural activity in parietal EEG sources and variations in LD, HE and steering response. Right parietal alpha activity was positively correlated with LD. This result indicates that participants deviated farther from the center of the lane when pre-stimulus alpha power was high, confirming its negative relationship with subsequent behavior (Hanslmayr et al., 2011). This fluctuation did not change significantly over time, indicating that participants were very good at maintaining the central task requirement. Combined, these results support the interpretation that the right parietal regions are involved in spatial processing and orienting, and when inactive (as indexed by high alpha power), driving performance is impaired as indexed by increased LD.

Simultaneously, the brain activity in the left parietal EEG sources may support ongoing assessment of HE and preparation for a corrective steering wheel movement that minimizes RT. The models for these three driving metrics found a main effect of time on task, and this may reflect changes in control over time suggesting that perhaps the control strategy changes to allow larger HEs that are met with larger steering corrections and slower RTs to ensure accuracy in the central task. The control strategy among these measures may be attributable to learned skills in participants who have driven for 10–20 years (age range 27–39) as evidenced by the lack of interaction between parietal activity and time on task for any of these driving metrics. More generally, these results suggest that left parietal alpha activity tracks control parameters necessary for steering corrections in support of the task objective. From a practical perspective, this research provides a framework within which one can consider implementing a driver error detection system. With the advent of autonomous vehicles that require strategic transfer of control between a human driver and an autonomous system, our findings suggest that monitoring brain activity may identify time frames when human error is more likely and trigger the shift in control from the human to the autonomous system.

Our results encapsulate theoretical predictions from two typically disparate areas of research. The compensatory control hypothesis within the psychology literature posits that central task requirements are preserved, even if participants must utilize more variable, often less efficient strategies (Hockey, 1997). Similarly, the uncontrolled manifold hypothesis in motor control theory posits that the primary performance criterion is prioritized, but variance is permitted in any variable that has redundant degrees of freedom (Scholz and Schöner, 1999). The core prediction of both theories was reflected in our results where participants allowed increased variation in HE, RT, and steering response while LD remained constant. Although nuances between the two theories exist, they both describe the human as a system which attempts to maintain critical task performance by permitting variability in redundant dimensions, and in our study, we identify differential neural substrates in the parietal cortex that may subserve these functions by using alpha to dynamically allocate attention to track sensory parameters relevant for successfully executing a visual-motor task.

## Author Contributions

JRB analyzed the data and wrote the majority of the manuscript. JOG assisted with the analysis, presentation of the data and writing. SEK and JMV also wrote and assisted with theoretical interpretation.

## Conflict of Interest Statement

The authors declare that the research was conducted in the absence of any commercial or financial relationships that could be construed as a potential conflict of interest.

## Footnotes

^1^http://sccn.ucsd.edu/eeglab/
